# Altered levels of interleukins and neurotrophic growth factors in mood disorders and suicidality: an analysis from periphery to central nervous system

**DOI:** 10.1038/s41398-021-01452-1

**Published:** 2021-06-02

**Authors:** Bharathi S. Gadad, Javier Vargas-Medrano, Enrique Ivan Ramos, Katherine Najera, Matthew Fagan, Angelica Forero, Peter M. Thompson

**Affiliations:** 1 Department of Psychiatry, Paul L. Foster School of Medicine, Texas Tech University Health Science Center, El Paso, TX 79905 USA; 2 Southwest Brain Bank, Texas Tech University Health Science Center, El Paso, TX 79905 USA; 3 Center of Emphasis in Cancer, Department of Molecular and Translational Medicine, Texas Tech University Health Sciences Center El Paso, El Paso, TX 79905 USA; 4 Department of Molecular and Translational Medicine, Paul L. Foster School of Medicine, Texas Tech University Health Sciences Center El Paso, El Paso, TX 79905 USA; 5 Department of Psychiatry and Behavioral Sciences, University of Texas Health Sciences Center, Houston, TX 77054 USA; 6 Division of Surgical Oncology, Department of Surgery, West Virginia University, Morgantown, WV 26506 USA

**Keywords:** Depression, Diseases

## Abstract

Interleukins and neurotrophins levels are altered in the periphery of patients with major depression and suicidal behavior, however it is not clear if similar abnormalities occur in the central nervous system. Our objective was to examine the association of IL-6, IL-1β, BDNF, and GDNF levels between postmortem plasma, cerebrospinal fluid (CSF), and brain tissue in a heterogeneous diagnostic subject groups including normal controls, mood disorders only, mood disorders with AUD/SUD (alcohol abuse disorder, substance abuse disorder), and AUD/SUD without mood disorders. To address these questions we collected postmortem plasma (*n* = 29), CSF (*n* = 28), and brain (BA10) (*n* = 57) samples from individuals with mood disorder, mood disorder with AUD/SUD, AUD/SUD and normal controls. These samples were analyzed using a multiplex based luminex assay with a customized 4-plex cytokine/interleukins- IL-6, IL-1β, BDNF, and GDNF human acute phase based on xMAP technology platform. Protein levels were determined using a Luminex 200 instrument equipped with Xponent-analyzing software. We observed IL-6 (*p* = 2.1e-07), and GDNF (*p* = 0.046) were significantly correlated between brain and CSF. In addition, IL-6 (*p* = 0.031), were significantly correlated between brain and plasma. Overall diagnostic group analysis showed a significant difference with brain GDNF, *p* = 0.0106. Pairwise comparisons showed that GDNF level is—39.9 ± 12 pg/ml, *p* = 0.0106, was significantly higher than in the brains derived from mood disorders compared to normal controls, —23.8 ± 5.5 pg/ml, *p* = 0.034. Brain BDNF was higher in suicide (*p* = 0.0023), males compared to females (*p* = 0.017), and psychiatric medication treated vs. non-treated (*p* = 0.005) individuals. Overall, we demonstrate that blood IL-6, GDNF and BDNF could be informative peripheral biomarkers of brain biology associated with mood disorders, substance disorders, and suicide.

## Introduction

Understanding the pathophysiology of mood and substance use disorders will involve integrating multiple biological pathways. Two areas of growing interest are the inflammation/interleukin and cell growth/neurotrophic factor cellular function pathways. Neurotrophic factors are involved in cellular proliferation, migration, differentiation, neuronal survival, as well as functional and structural maintenance of neurons in the adult brain^1,2^. Brain derived neurotrophic factor (BDNF) and glial cell line-derived neurotrophic factor (GDNF) are crucial brain signaling proteins whose deficiency in key neural pathways has been associated with the development of psychiatric disorders^1,3^. BDNF and GDNF have also been postulated to have a role in alcohol dependence, withdrawal and regulation of alcohol consumption^2–4^.

Interleukins are responsible for promoting cell growth, differentiation, functional activation, and play a crucial role in mediating the interaction of inflammatory and immune cells^5^. The role of interleukins, neurotrophic factors and immune dysregulation has been studied in many psychiatric disorders and suicide^6,7^. Aberrant levels of proinflammatory interleukins have been reported in major depressive disorder (MDD), schizophrenia, bipolar disorder (BD), alcohol use disorder (AUD), suicidality and substance abuse disorder (SUD)^8–11^.

Interleukins such as IL-1β, IL-6, and TNF-α become elevated in the brain during chronic stress and depression. Meta-analyses for MDD, schizophrenia, and BD all found elevated blood levels of interleukin (IL)-1β, soluble interleukin-2 receptor (sIL-2R), and tumor necrosis factor (TNF)-α^8,12,13^. Another meta-analyses found elevated blood levels of IL-6 in MDD^14^. An abnormality in interleukins may also be associated with suicidal behavior and this observation is supported by a study from Tonelli et al.^10^. They found increased mRNA expression of IL-4 and IL-3 in the PFC (prefrontal cortex) of women and IL-13 in men who died by suicide compared with normal control subjects^10^. Janelidze et al. determined the levels of interleukins in plasma of suicidal and non-suicidal depressed patients and found that the levels of proinflammatory interleukins IL-6 and TNF-α were significantly higher in suicide attempters compared with non-suicidal depressed patients^15^. A qualitative review of inflammatory changes associated with suicidal behavior reported that IL-2, IL-6, IL-8, and TNF-α and BDNF were the most commonly altered inflammatory makers^10,16^. The peripheral findings can arise from local production in the central nervous system (CNS) or translocation across the BBB from the periphery^17–20^.

In this study to better understand the relationship between peripheral (plasma) and CNS (brain and CSF) levels of neurotrophic factors and interleukins, we measured IL-6, IL-1β, BDNF, GDNF in postmortem brains, cerebrospinal fluid (CSF), and plasma from the same individuals who had psychiatric disorders. We then looked at the levels of these proteins in brain, CSF, and plasma between the major psychiatric diagnoses.

## Materials and methods

### Sample collection

- *Postmortem brain samples*: The Southwest Brain Bank (SWBB), Department of Psychiatry at Texas Tech University Health Sciences Center obtained consent for tissue donation from the next-of-kin. The research protocol has been reviewed by the Institutional Review Board (IRB) at TTUHSC-El Paso and at the University of Texas Health San Antonio. The IRB Committee, Texas Tech University Health Sciences Center, El Paso have reviewed and approved the research protocol for Southwest Brain Bank for the collection of human postmortem subjects, CSF, and plasma samples for the study. Postmortem brain tissue, CSF, and plasma were obtained from each donation. To preserve fresh tissue the brain was cut into 1 cm-thick blocks, flash frozen in isopentane dry ice slurry and stored at −80 °C; CSF was aliquoted and stored at −80 °C. Brodmann’s area (BA) 10 was identified and dissected from frozen blocks.
- *Cerebrospinal fluid*: CSF samples were obtained by lumbar puncture from the L4–5 or L3–4 interspace during the postmortem tissue brain extraction. CSF samples were immediately placed on ice and centrifuged at 4000 × *g*. Supernatants were aliquoted and stored at −80 °C until assays were performed.
- *Plasma samples*: 10 ml peripheral samples were collected in EDTA tubes. Samples were centrifuged at 1200 rpm at room temperature for 15 min to isolate plasma. Extracted plasma was aliquoted and stored at −80 °C until further assay.

The total number of subjects used in the study, are 57 postmortem brain subjects, 29 subjects had brain and CSF, and 28 had brain and plasma from the same person. In 8 of the 57 subjects, brain, CSF and plasma were from all subjects. The samples were de-identified and blinded throughout the experimental analyses.

### Clinical characterization

Clinical information was obtained from medical records and next-of-kin interviews about the donor. The M.I.N.I version VII following DSM IV TR criteria (Diagnostic and Statistical Manual for Mental Disorders edition IV TR) and the Structured Clinical Interview for DSM IV TR—Personality Disorder (SCID-IV TR PD) were used to make the psychiatric diagnosis^21^. All interview materials, demographic materials, medical/psychiatric histories, toxicology, neuropath, and autopsy reports are presented, for a diagnosis using consensus review of obtained information by expert psychiatric diagnosticians (M.D. and Ph.D.). For this study we separated the samples into normal controls (NC) without any psychiatric diagnosis, Mood Disorders (major depression and BD without comorbid alcohol/substance use disorders), Mood Disorder with comorbid alcohol and substance use disorders—Mood/AUD-SUD and AUD/SUD only groups.

#### Case selection: inclusion and exclusion criteria

*Inclusion criteria*: (1) Evidence of major mental illness, or lack of evidence (controls), (2) consent of the donor or NOK for tissue donation and agreement to participate in clinical interview, (3) postmortem Interval (PMI) <48 h, and (4) age between 18 years old and 100 years old. *Exclusion criteria*: (1) Decomposed/PMI above 48 h, (2) Chronic non-alcohol withdrawal induced seizures (unless seizures are focus of research), (3) On respirator ventilation >72 h, (4) damage to the brain caused by closed head injury or other trauma sufficient to render the tissue not usable i.e., bullet wound, (6) Large brain hemorrhages, infarcts (tissue from the contralateral side may be taken if the neuropathologist determines the infarct is a small lacunar type), tumors or other intracranial lesions that disrupt normal brain structure, (7) known viral infection, such as HIV, Hepatitis A, B, or C, SARS, (8) bacterial infection, such as bacterial encephalitis, meningitis, (9) major sepsis, and (10) Prion disease: the case selection are made based on the discretion of Director of the brain bank, that donation is not in the best interest of the SWBB and TTUHSC-El Paso.

### Multiplex analyses of interleukins and neurotrophins

Brain, CSF, and plasma samples were analyzed with a multiplex assay using a customized 4-plex IL-6, IL-1β, BDNF, and GDNF human acute phase panel based on xMAP technology platform. Testing used a customized magnetic human premixed multi analyte luminex kits for IL-6, IL-1β, BDNF, and GDNF, purchased from R&D Systems (Minneapolis, MN, USA). Sample Lysis Buffer was prepared using 100 mL dH_2_O; 0.79 m MTris HCL (50 mM) (pH 7.5); 0.044 mM EDTA (2 mM); 1 μL protease inhibitor (Sigma, Co).

Protein samples and standards were diluted in PBS containing 1% bovine serum albumin (BSA, Sigma-Aldrich; St. Louis, MO) and conjugated MagPlex beads were always diluted in PBS with 1% BSA and Complete Mini. Samples were placed in plates were read on a Luminex 200 platform (Luminex Corp, Austin TX) running xPONENT 3.1 to determine protein analyte concentrations according to the manufacturer’s protocol. Prior to adding conjugated MagPlex beads to samples, beads were thoroughly resuspended by vortexing and sonication for 20 s each. Diluted samples and standards were then loaded onto a black flat-bottom 96-well plate (Greiner; Monroe, NC), mixed with resuspended magnetic beads at a concentration of 2500 beads per well, and incubated overnight. This and all following incubation steps were performed with she samples on a shaking device in the dark and at room temperature. Then, the beads were washed twice and then incubated for 3–4 h with 100 ng of biotinylated antibody (in PBS with 1% BSA) per well. After that, beads were washed twice and then incubated for 1 h with 1 μg of phycoerythrin conjugated streptavidin per well (Life Technologies; Carlsbad, CA). After that beads were washed twice, resuspended in PBS, incubated for 5 min, and then read using the Luminex 200 instrument. Interleukins and neurotrophins detected along with assay range, were measured in pg/mL using Luminex software and interpreted only if the intra-and inter-assays coefficients of variation were <10% of detection limits (or precision range) specified by manufacturer. The concentration of proteins are expressed in pg/mL.

### Statistical analysis

All experimental and basic patient data are shown as mean ± standard deviation. Categorical differences between groups such as gender, smoking, family psychiatric history was tested using the Chi-square test. We used IBM SPSS Statistics version 25 and R version 3.6. To correlate the levels of overall interleukins and neurotrophins across all samples types from periphery to CNS (brain, CSF, plasma) we performed a multivariate regression analysis using co-variates of PMI, RIN and pH on the transformed data. In a subgroup, *N* = 8 where brain, CSF and plasma was obtained from the same individual correlations were conducted a confirmatory correlation analysis using Spearman’s correlation coefficient. Alpha was set at 0.05. To determine differences in the levels of IL-6, IL-1β, BDNF, and GDNF between diagnostic groups we used the Kruskall–Wallis test for overall testing. If the result was significant Mann-Whitney U test was used for pairwise comparisons. Correlation was calculated in R statistical software (version 4.0.3) using spearman parametric correlation test between cytokine/interleukins (IL-6, IL-1β, BDNF, and GDNF) and postmortem demographics in brain, CSF, and plasma. Supplementary Tables with the correlation coefficients and significance *p*-values were computed for each group (brain, CSF, and plasma).

### General linear model

A general linear model was generated in R software environment using the lm function by fitting a model with GDNF (concentration) predicted by group (control or mood disorder). Analysis of Variance (ANOVA) table was calculated in R using the fitted model. The model was generated with the summary function in R displaying the estimates, standard errors, residual standard error, and multiple R-squared values. Lastly, a set of four plots were generated in R for residuals versus fitted values, a Q–Q plot of standardized residuals, a scale-location plot (square roots of standardized residuals versus fitted values), and a plot of residuals versus leverage.

## Results

### Demographic information

The demographic and clinical characteristics for clinical groups are listed in Table 1. Of the 57 subject material used, 29 had brain and CSF, and 28 had brain and plasma from the same person. In 8 of the 57 subjects, brain, CSF and plasma were from all subjects. The demographic information are based on the subjects from postmortem brain, plasma and CSF of Mood Disorders, Mood/AUD-SUD and AUD/SUD with NC. The number of subjects and the data related to RIN, pH, PMI, Gender, Ethnicity and Cause of Death from the postmortem brain subjects are in Table 1.

**Table 1:** Demographic information of the subjects from postmortem brain, plasma, and CSF of Mood Disorders, Mood/SUD, and AUD/SUD with Normal Controls.

Category	Normal control *N*	Mood disorders *N*	Mood disorders with SUD *N*	AUD/SUD *N*
Gender				
Male	14	5	6	11
Female	4	4	11	2
Ethnicity				
Hispanic	15	1	5	7
Anglo	3	7	12	6
Smoking				
Yes	5	6	10	8
No	13	3	7	5
Suicide				
Yes	0	2	6	2
No	18	7	11	10
Family Psych Hx				
Yes	5	5	8	6
No	11	4	9	7
Psych. Meds				
Yes	0	5	7	11
No	18	9	10	2
Age	63.2 ± 14	56.3 ± 7	45.7 ± 12	50 ± 11
RIN	6.3 ± 0.1	6.7 ± 1.6	7.5 ± 1.3	6.8 ± 1.6
pH	6.2 ± 0.2	6.3 ± 0.3	6.3 ± 0.3	6 ± 0.4
PMI-Hr	28.3 ± 8	24.6 ± 8	30 ± 7.4	26.5 ± 7

### Interleukins and neurotrophins in brain, CSF, and plasma

**Fig. 1:**
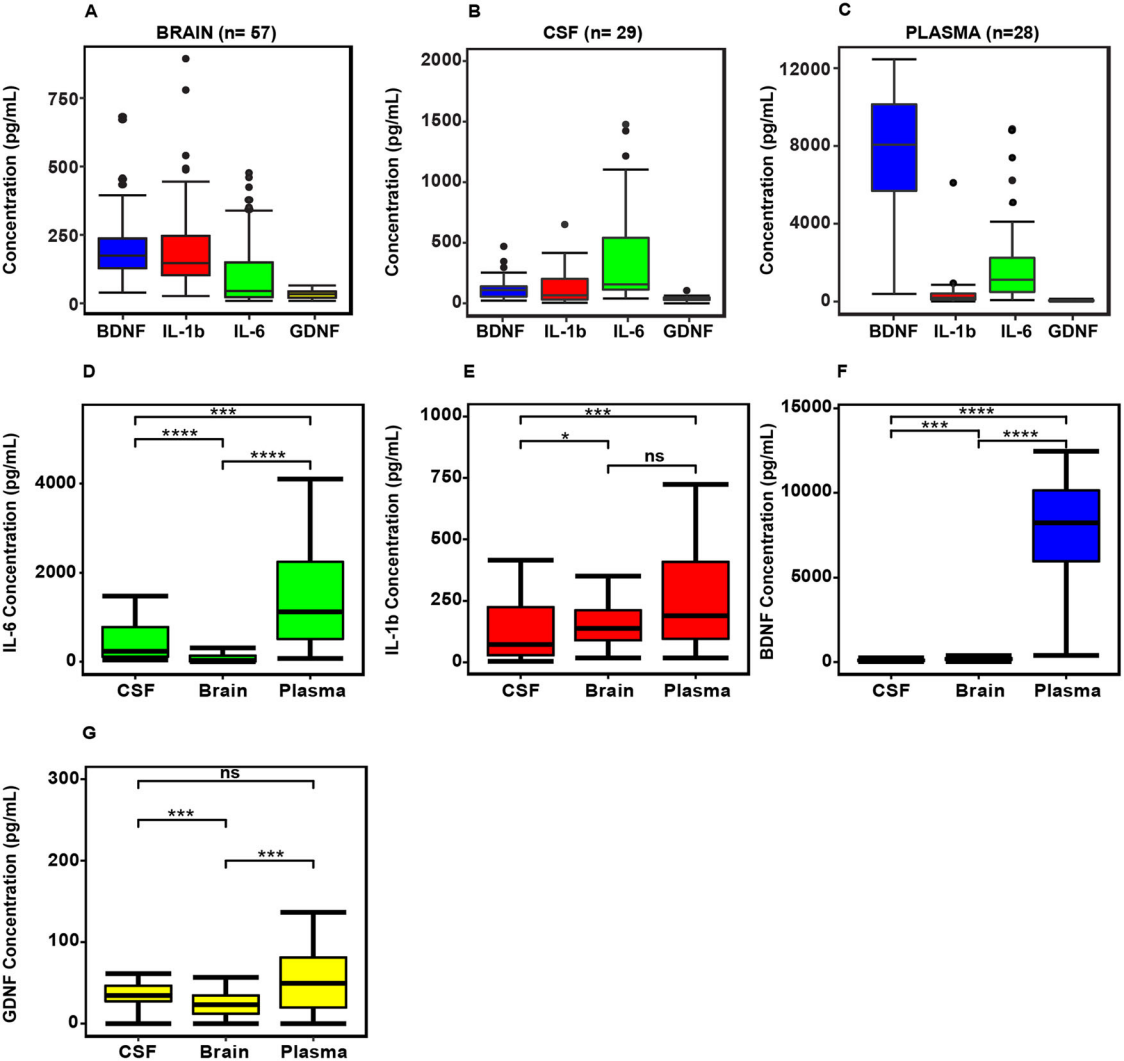
Protein levels of interleukins (IL-6 and IL-1β) and neurotrophins (BDNF, GDNF) in the postmortem brain (n = 57), CSF (n = 29), and plasma (n = 28) (**A**–**C**). Concentration of IL-6, IL-1β, BDNF, and GDNF in brain, plasma and CSF between (**D**–**G**). Asterisk indicates *p < 0.01; **p < 0.06.

Figure 1, reveals the overall concentration of IL-6, IL-1β, BDNF, and GDNF in the brain, CSF, and plasma. The interleukins and neurotrophins showed a differential pattern with GDNF being lower than IL-6, IL-1β, and BDNF in all compartments (Fig. 1A–C). The data is re-plotted showing the concentration of brain, plasma and CSF between IL-6, IL-1β, BDNF, and GDNF (Fig. 1D–G). The plasma levels of IL-6, IL-1β, BDNF, and GDNF are shown in the Supplementay Table 1A. Interleukins and Neurotrophins CSF/Brain ratio and Plasma/Brain ratio is shown in Supplementary Table 1B. The CSF/Brain ratio for IL6, Il-1β, BDNF, and GDNF were 8.6, 0.7, 0.48 and 1.5, respectively. However, Plasma/Brain ratio for IL6, Il-1β, BDNF, and GDNF are 37.3, 5.0, 35.9, and 2.9. Overall, interleukins and neurotrophins showed differential levels of expression across the brain, CSF, and plasma compartments tested.

### Correlation of interleukins and neurotrophins from periphery to CNS

**Fig. 2:**
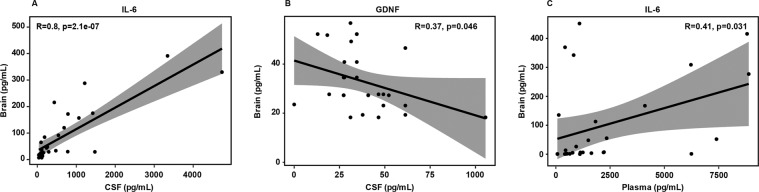
Correlation plots of interleukins and neurotrophins in Brain, Plasma, and CSF. IL-6 (*p* = 2.1e-07) were significantly altered in Brain vs. CSF (**A**), GDNF (*p* = 0.046) are significantly altered in Brain vs. CSF (**B**). IL-6 (*p* = 0.031), are significantly altered in Brain vs. Plasma (**C**).

We conducted statistical multivariate regression analyses of all subjects grouped together to correlate the levels of interleukins and neurotrophins across all samples types from periphery to CNS (plasma, *n* = 28; CSF, *n* = 29; and brain, *n* = 57). We observed IL-6 (*p* = 2.1e-07), and GDNF (*p* = 0.046) were significantly correlated between brain and CSF (Fig. 2A, B). In addition, IL-6 (*p* = 0.031), were significantly correlated between brain and plasma (Fig. 2C). There was a trend to significance with GDNF brain to plasma correlation, *p* = 0.05 (data not shown).

In a sub-analysis we considered the, eight subjects where we obtained postmortem brains, CSF, and plasma, from the same individual. In this group, brain IL-6 was correlated to CSF IL-6 (*p* = 0.004), and plasma IL-6 (*p* = 0.05). Brain IL-1β correlated to plasma IL-1β (*p* = 0.037). Spearman’s correlations were computed between interleukins/neurotropic growth factors (IL-6, IL-1β, BDNF, and GDNF) and postmortem demographics (age, sex, PMI, pH, RIN, and cause of death) in postmortem plasma, CSF, and brain tissue. Brain results (Supplementary Table 2) shows six statistically significant (*p* < 0.05) correlations of which two are positive (IL-6-IL1β, BDNF-GDNF) and four are negatively correlated (IL-6-pH, Il-6-RIN, BDNF-Gender, and GDNF-Age). Results for CSF (Supplementary Table 2) pinpoint ten statistically significant (*p* < 0.05) correlations with five positive correlation (Il-6-Il-1β, GDNF-Il-6, IL1β-GDNF, GDNF-gender, and race), while five reveal negative correlation (IL6-pH, IL1β-pH, IL-1β-RIN, cause of death and GDNF-RIN). Lastly, plasma data (Supplementary Table 2) illustrate seven statistically significant (*p* < 0.05) correlations with two are positively correlated (BDNF-pH, GDNF-1L-1β) on the other hand, five negatively correlated (IL-1β-Age, pH, IL-6-pH, RIN, and BDNF). As discussed in the previous data, there were no correlations with CSF and plasma with interleukins and neurotrophins. Taken together, the data suggest positive/negative statistically significant correlations between the different cytokines/interleukins with postmortem demographics from periphery to CNS.

### Altered levels of interleukins and neurotrophins based on clinical categories

**Fig. 3:**
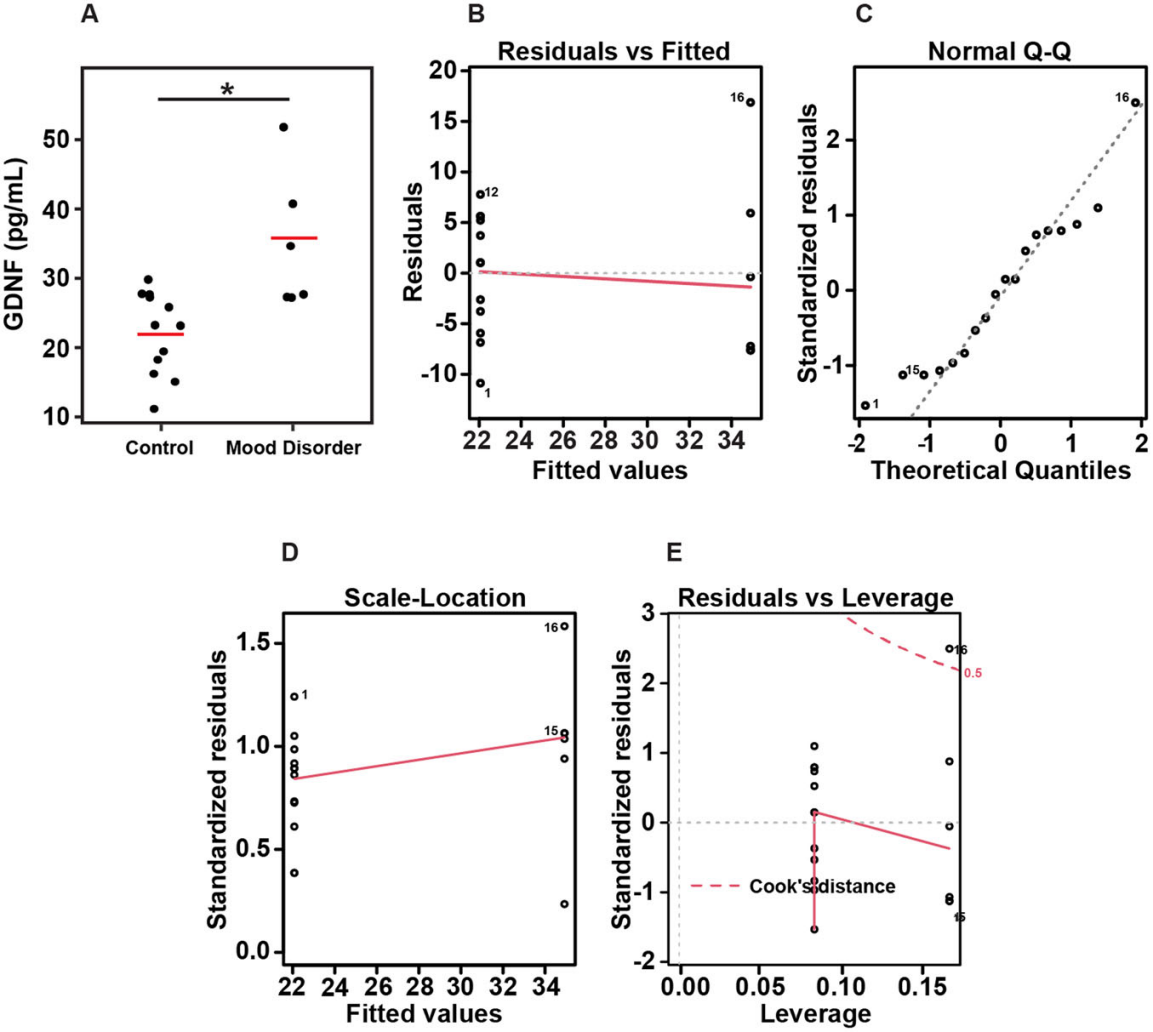
Box plots of GDNF presented based on the clinical diagnostic groups. Diagnostic group analysis from control vs mood disorder showed a significant difference with brain (BA10) GDNF, *p* = 0.0106. **A** Actual values of the samples with the linear model, **B** residuals versus fitted values, **C** Q–Q plots of standardized residuals, **D** scale-location plot, and **E** residuals versus leverage plot.
iii. *Interleukins and neurotrophins in suicide and non-suicide causes of death*: We separated the subjects into those that died by suicide and those that did not. We found brain BDNF levels in suicide 352 ± 203, was significantly higher compared to non-suicide—241 ± 129, *p* = 0.0023 (Fig. 4A).

i. *Interleukins and Neurotrophins in diagnostic groups*: The brain–CSF subjects’ assays were run at a different time than the brain–plasma group and analyzed separately. In the brain–CSF samples PMI and in the brain–plasma group ethnicity and smoking were different between groups, Supplementary Table 3. In the brain–plasma diagnostic groups were matched for age, gender, PMI, RIN, and pH. The overall diagnostic group analysis showed a significant difference with brain GDNF, *p* = 0.0106. Pairwise comparisons showed that GDNF level—39.9 ± 12, *p* = 0.0106, was significantly higher in the brains from mood disorders compared to normal controls, NC-23.8 ± 5.5, *p* = 0.034.
ii. Figure 3 shows the graphical representation of the linear model. Figure 3A shows the actual values of the samples with the linear regression. The general linear model results were generated using the formula for the model GDNF (concentration) is predicted by group (control or mood disorder), omitting the intercept. In our model, the difference between the observed and predicted values (residuals) are symmetrically distributed (min and max, as well as 1Q and 3Q), while the median value is close to zero. The estimates used to predict the value of the response variable show a projected mean of 22.060 (pg/mL) GDNF concentration for the control group, while for a patient in the MDD group, we expect the mean GDNF concentration to be 12.84 (pg/mL) higher. The standard error (2.137 and 3.022 for control and MDD, respectively) depicts the average amount that the estimate varies from the actual value. *T* values (Estimate/Std. Error) of 10.32 and 11.55 were obtained for control/MDD, and *p* values for the *t* test determined the coefficients are significant. Moreover, the quality of the linear regression fit was measured by the residual standard error of 7.402, an R-squared value of 0.9375 displays how well the model fits the actual data, and an F-statistic of 120, as well as a *p* value of 2.331e−10 show the model as a whole is statistically significant. Figure 3B–E shows the residuals versus fitted values (3B) Q–Q plots of standardized residuals (3C), Scale-location plot (3D), and Residuals versus leverage plot (3E).

**Fig. 4:**
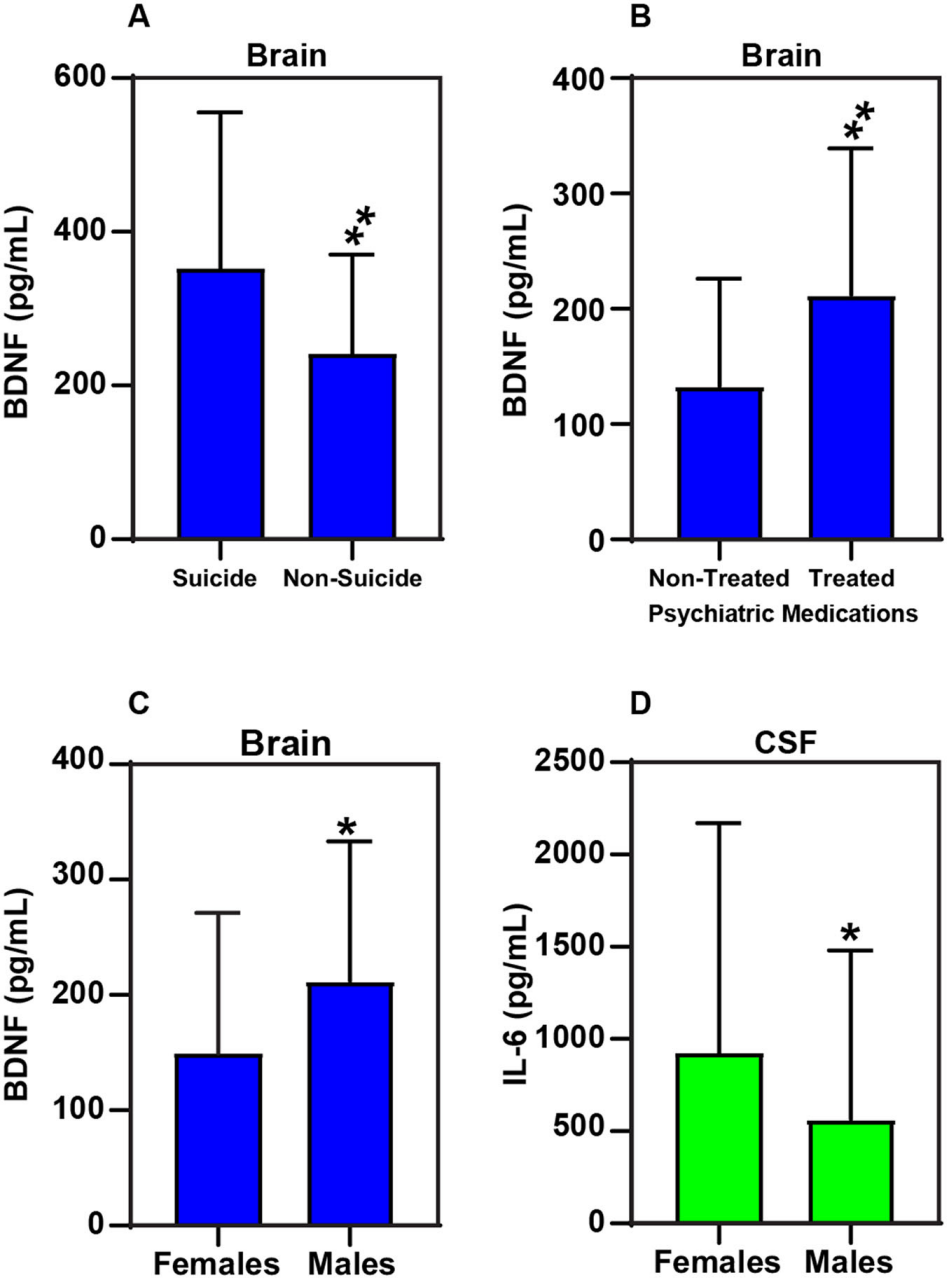
Alterations in the levels of IL-6 and neurotrophins (BDNF) based on gender, suicidality, and psychiatric medications. **A** Brain BDNF was elevated in suicide—3 ± 203, compared to non-sucide—2 ± 129, *p* = 0.0023. **B** Brain BDNF levels without psychiatric medications were significantly lower 133 ± 93 compared to the treated subjects 212 ± 127, *p* = 0.005. **C** Brain BDNF were significantly lower 149 ± 122 in females compared to males 212 ± 121, *p* = 0.017. **D** CSF IL-6 levels were significantly higher in Females 923 ± 1246 compared to males 565 ± 915, *p* = 0.021.
iv. *Interleukins and neurotrophins with psychiatric medication use*: All the affected subjects were divided by the use of psychotropic medication. We found brain BDNF levels without psychiatric medications—133 ± 93 were significantly lower than treated subjects 212 ± 127, *p* = 0.005 (Fig. 4B). Changes in the level of Brain BDNF based on with and without psychiatric medications (Supplementary Fig. 1) suggesting a gender difference in terms of drug effect.
v. *Interleukins and* *neurotrophins with sex*: we looked at the affected subjects only and found the brain BDNF in females was 149 ± 122 and significantly lower than males 212 ± 121, *p* = 0.017 (Fig. 4C). CSF IL-6 was significantly higher in females 923 ± 1246 compared to males 565 ± 915, *p* = 0.021 (Fig. 4D). Supplementary Fig. 2 shows gender distribution between males and females across the diagnostic groups normal control, Mood/SUD, Mood, and AUD/SUD.

## Discussion

**Fig. 5:**
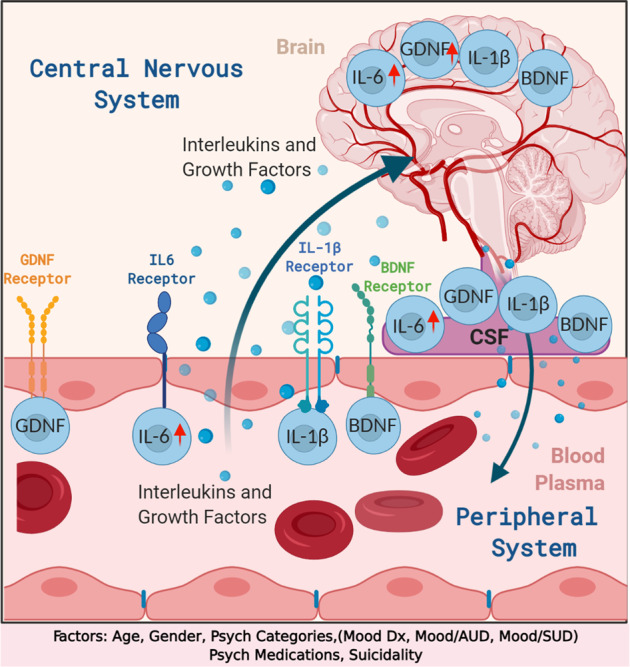
Overview of the study to understand the interlink between central nervous system and periphery in individual subjects. Graphical illustration of main top-down, central nervous system (CNS)–Brain/CSF to periphery, and bottom-up (periphery to CNS) communication pathways through the receptors and summary of peripheral and central pathology through interleukins—IL-6 and Il-1β and neurotrophins, BDNF, and GDNF. Factors that are associated with the analyses are suicidality, age, gender, psychiatric medications, RIN, pH, and PMI.

The present study is the first to look at the levels of interleukins Il-6, IL-1β; neurotrophins BDNF, GDNF in human postmortem CSF, plasma, and brain from the same individuals. Moreover this study reports the alteration in the levels of interleukins and neurotrophic factors across periphery and CNS between Mood disorder, mood disorder with AUD/SUD, compared to control subjects. Also we report in the study the differential levels of interleukins and neurotrophins based on gender, suicidal vs. non-suicidal and psychiatric medications across periphery and CNS. Figure 5, depicts the details and the overview of the study, based on our major findings to understand the interlink between CNS and periphery in individual subjects which supports a psychoneuroimmunological understanding of mental illness pathology. Graphical illustration of main top-down, CNS–Brain/CSF to periphery and bottom-up (periphery to CNS) communication pathways through the receptors and summary of peripheral and central pathology through interleukins—IL-6 and Il-1β and neurotrophins, BDNF and GDNF. However the increased permeability of blood–brain barrier (BBB) in depression is high enough to let the neurotrophic factors and interleukins through specific receptors passing through BBB. The interleukins and neurotrophins produced in the periphery might pass through the dysregulated BBB into the CNS. Also, altered levels of IL-6 and GDNF in CSF and brain might be, at least partially, due to the restorative effect to the dysregulated BBB function. The present study is novel to the literature depict the role of interleukins in periphery and CNS in the same participants. Whenever using blood interleukins or neurotrophins to understand the biology of mental illness, the question arises if the results reflects what’s occurring in the brain? BDNF in rodents has been studied and authors reported a correlation between brain and periphery^22^. But there is no direct study of brain and blood in humans. First we looked at this question by determining if there was a correlation of interleukins and neurotrophins levels between brain, CSF and plasma. To our knowledge the relationship between brain–CSF and plasma has never been directly shown in humans. Here we report for the first time the relationship between IL-6, IL-1β, BDNF, and GDNF from periphery to CNS using plasma, CSF and human postmortem brain. In the total sample we found IL-6, BDNF, and GDNF were significantly correlated between brain and CSF. IL-6, BDNF, and GDNF were also correlated between brain and plasma. Analyses from the, brain, CSF and plasma were from the same subjects, revealed a strong IL-6 correlation between brain–CSF–plasma. Surprisingly in this subgroup we also saw a significant correlation between brain IL-1β and plasma IL-1β which was not seen when all subjects were studied together.

These correlation findings may arise from a normal physiological peripheral-CNS relationship or a consequence of the breakdown of the BBB after death. Past research has demonstrated that regulated interleukins pass the BBB^23,24^. However the role of neurotrophins still remains unclear. GDNF is reported to not pass the BBB^23^. With BDNF there are reports demonstrating passage and others that show it cannot pass the BBB^22–25^. Based on these reports one would expect there to be differential levels of IL-6, IL-1β, BDNF, and GDNF between the brain, CSF and plasma. Which is what we find with our data. Suggesting the BBB was present and our findings are not an artifact of necrotic tissue.

GDNF is expressed abundantly throughout the brain, it is known to play a crucial role in the development and maintenance of glial cells, serotonergic and dopaminergic neurons^1,4,26^; it also regulates noradrenergic and GABAergic pathways. GDNF is also known to protect both neurons and glia cells against oxidative stress^27^. Up-regulation of GDNF by astrocytes and microglia may be a protective mechanism to restrain neuronal loss observed in different types of brain diseases. Here we observed GDNF level was significantly greater than NC in the mood disorders group. Prior studies have looked at GDNF in serum and found the level varies between different psychiatric diagnoses. Higher levels are reported in mood disorders, AUD and ADHD^26,28–31^. Zhang et al. did not find any effect of medication in GDNF levels^32^. Conversely, data from in vitro studies demonstrate increased levels of GDNF in the presence of antidepressants and mood stabilizing agents^1,4,27^. The majority of individuals were on certain psychiatric medications including mood stabilizers and antidepressants. With the number of individuals used in this study we are not able to parse out what influence if any medication treatment had on our result.

BDNF mediates neuronal plasticity, migration, survival, and crosses the BBB In the brain BDNF is produced by both neurons and glia. Based on our results from the present study, we found strong correlations of BDNF with brain and CSF and brain and plasma. This finding is consistent to BDNF findings in non-humans^22^. We also show that in individuals who killed themselves, the brain BDNF level was higher than individuals who died by other causes. Prior studies shows lower levels of BDNF in postmortem prefrontal cortex^33^ and hippocampus^19^ of brains from subjects with MDD and suicide. However not all studies shows this. In another postmortem brain study of individuals with a mood disorder of which half died by suicide, the authors reported no significant BDNF difference between depressed and control cohorts^34^. In the periphery, a large study did not identify any BDNF level differences in suicide^35^. Our results are partly in accordance with Eisen et al.^35^ where they did not find significant difference in BDNF levels in attempted suicide subjects.

In our study we showed individuals with psychopharmacological treatment had higher brain BDNF than those not receiving treatment. Several prior studies have shown antidepressant use may increase peripheral BDNF levels^9,19,22,36^. Six individuals in the affected group were on a serotonin reuptake blocker antidepressant at the time of death. Given the nonuniformity of prior research there is not a clear association of BDNF suicide. However, the finding of increased GDNF in mood disorders and increased BDNF with antidepressant treatment supports a role of growth factors in mood disorder or its medication treatment.

Interleukins are regulatory peptides that participate in the host defense and repair processes of tissues^7^. The interleukins found in the brain that are most studied are IL-1β, IL-6, and TNF-α. Among their many roles, they are known to modulate neuroendocrine functions, sleep and in neuroinflammation. They are also strongly implicated in the neurobiology of mood and substance disorders^15,37–40^. Literature in this area would indicate that both IL-6 and IL-1β would be elevated in the affected groups. In our study there were no statistically significant difference in IL-6 and IL-1β between NC and the affected groups.

Overall, the present study explored the correlation between brain–CSF–plasma and the differences in levels of interleukins and neurotrophic neurotrophins in periphery to CNS in a clinical sample; our major findings showed that peripheral GDNF, BDNF, IL-6, and most likely IL-1β levels correlates with their concentration in the CNS across the BBB to the plasma. We show the brain level of GDNF is higher for the subjects with a mood disorder compared to the control group and brain BDNF is higher in the treated groups. Although there is no direct evidence to prove inverse correlation in the levels of GDNF from brain to periphery. However the increased permeability of BBB in depression is high enough to let the GDNF passing through BBB. It might result from that the GDNF produced in the periphery would pass through the dysregulated BBB into the CNS to repair the impaired neurons in patients with depression. Also, the altered levels of GDNF in CSF might be, at least partially, due to the restorative effect of the GDNF to the dysregulated BBB function. The present study is novel to the literature to demonstrate the role of disease marker of peripheral and CNS levels of GDNF in depression and MDD and its role with antidepressant treatment. Hence, the interaction between dysregulation of BBB and neurotrophic levels of GDNF might play a role in the pathogenesis of depression. Together these findings indicate that increased GDNF could be a compensatory response that might appear in some MDD subjects. However, there are some limitations in our study. All subjects with a psychiatric diagnosis that are investigated in this study were receiving pharmaco-treatment (mood stabilizing agents, antipsychotics, and/or antidepressants) and it is not possible to exclude the effect of drugs on the levels of interleukins and neurotrophic factors. The other limitation is that the mood disorders groups had individuals who had either major depression or BD. So the finding is not specific to a single psychiatric disorder. The overall number of subjects and also the findings of these changes in the levels of these inflammatory markers from the same individual’s plasma, CSF and postmortem brain subjects included in the present study and the strict inclusion criteria must be considered strengths of our work, we have considered co-variates such as RIN, pH, and PMI for the postmortem brain subjects.

## Supplementary information

Supplementary Figure Legends and Tables

Supplementary Figure-1

Supplementary Figure-2
