# Supplementary material for: Altered levels of interleukins and neurotrophic growth factors in mood disorders and suicidality: an analysis from periphery to central nervous system
Source: Transl Psychiatry. 2021 Jun 2;11:341. doi: 10.1038/s41398-021-01452-1 (PMC8171230; doi:10.1038/s41398-021-01452-1)
Supplement: Supplementary file 1 — Supplementary Figure Legends and Tables [file 41398_2021_1452_MOESM1_ESM.docx]

**Supplementary Figure Legends**

**Supplementary Figure 1.** Scattered plot of Brain BDNF levels based on psychiatric medications (SSRI).

**Supplementary Figure 2.** Brain GDNF changes in males vs. females across the diagnostic groups.

**Supplementary Table 1, Data from N=8 subjects with brain, CSF and plasma from the same individual.**

**Table 1A**

|  | Interleukin | | Neurotrophins | |
| --- | --- | --- | --- | --- |
|  | **IL6** | **Il-1β** | **BDNF** | **GDNF** |
| Brain pg/ml | 92.2 | 183.1 | 187.6 | 22.0 |
| CSF pg/ml | 795.5 | 133.1 | 90.9 | 35.6 |
| Plasma pg/ml | 3436.1 | 917.3 | 6727.3 | 62.8 |

**Table 1B**

|  | Interleukin Neurotrophins | | | |
| --- | --- | --- | --- | --- |
|  | **IL6** | **Il-1β** | **BDNF** | **GDNF** |
| Brain pg/ml | 92.17875 | 183.06875 | 187.57875 | 21.99875 |
| CSF pg/ml | 795.4938 | 133.13875 | 90.88875 | 35.6325 |
| Plasma pg/ml | 3436.1 | 917.3 | 6727.3 | 62.8 |
| CSF/Brain Ratio | 8.6 | 0.7 | 0.48 | 1.5 |
| Plasma/Brain Ratio | 37.3 | 5.0 | 35.9 | 2.9 |

**Supplementary Table 2: Correlations between all postmortem demographics and IL-6, IL-1β, BDNF, and GDNF protein levels in the brain and periphery.**

|  | **Brain IL6** | **Brain IL1B** | **Brain BDNF** | **Brain GDNF** | **CSF IL6** | **CSF IL1Beta** | **CSF BDNF** | **CSF GDNF** | **Plasma IL6** | **Plasma BDNF** | **Plasma IL1Beta** | **Plasma GDNF** | **Smoking** | **Family**  **HX** | **Age** | **Gender** | **PMI** | **Race** | **pH** | **RIN** | **Cause Of Death** |
| --- | --- | --- | --- | --- | --- | --- | --- | --- | --- | --- | --- | --- | --- | --- | --- | --- | --- | --- | --- | --- | --- |
| **Brain IL6** | 1.000 | .653^**^ | -0.152 | 0.158 | .842^**^ | .674^**^ | 0.060 | .623^**^ | 0.439 | -0.414 | .607^**^ | 0.400 | -0.103 | 0.029 | 0.017 | 0.113 | 0.155 | 0.016 | -.606^**^ | -.439^**^ | -0.031 |
| **Brain IL1B** | .653^**^ | 1.000 | -0.175 | 0.253 | 0.233 | 0.087 | -0.107 | 0.208 | 0.014 | 0.046 | 0.363 | 0.241 | 0.047 | 0.307 | -0.123 | 0.164 | 0.132 | -0.306 | -0.220 | -0.180 | 0.009 |
| **Brain BDNF** | -0.152 | -0.175 | 1.000 | .496^**^ | -0.173 | -0.397 | 0.243 | -0.305 | -0.176 | 0.368 | -0.262 | 0.026 | -0.137 | 0.060 | 0.153 | -.463^**^ | -0.059 | 0.216 | -0.095 | 0.048 | -0.054 |
| **Brain GDNF** | 0.158 | 0.253 | .496^**^ | 1.000 | -0.019 | -0.280 | -0.054 | 0.196 | -0.485 | 0.454 | -0.160 | 0.130 | -0.195 | 0.091 | .423^*^ | -0.037 | 0.159 | 0.058 | -0.272 | -0.224 | 0.135 |
| **CSF IL6** | .842^**^ | 0.233 | -0.173 | -0.019 | 1.000 | .711^**^ | 0.152 | .665^**^ | NC | NC | NC | NC | -0.395 | -0.212 | 0.157 | 0.292 | -0.160 | 0.341 | -.641^**^ | -0.348 | -0.273 |
| **CSF IL1Beta** | .674^**^ | 0.087 | -0.397 | -0.280 | .711^**^ | 1.000 | 0.196 | .617^**^ | NC | NC | NC | NC | -0.250 | -0.347 | -0.051 | 0.399 | -0.203 | 0.273 | -.746^**^ | -.558^*^ | -.502^*^ |
| **CSF BDNF** | 0.060 | -0.107 | 0.243 | -0.054 | 0.152 | 0.196 | 1.000 | 0.297 | NC | NC | NC | NC | -0.212 | -0.077 | -0.345 | 0.107 | -0.068 | 0.228 | -0.088 | 0.264 | 0.011 |
| **CSF GDNF** | .623^**^ | 0.208 | -0.305 | 0.196 | .665^**^ | .617^**^ | 0.297 | 1.000 | NC | NC | NC | NC | -0.232 | -0.145 | 0.258 | .460^*^ | -0.324 | .469^*^ | -0.403 | -.479^*^ | -0.077 |
| **Plasma IL6** | 0.439 | 0.014 | -0.176 | -0.485 | NC | NC | NC | NC | 1.000 | -.598^**^ | 0.440 | 0.028 | 0.065 | -0.154 | -0.186 | 0.115 | 0.195 | 0.413 | -.615^**^ | -.506^*^ | -0.251 |
| **Plasma BDNF** | -0.414 | 0.046 | 0.368 | 0.454 | NC | NC | NC | NC | -.598^**^ | 1.000 | -0.137 | 0.088 | -0.371 | 0.115 | -0.021 | -0.346 | -0.375 | -0.269 | .473^*^ | 0.446 | -0.179 |
| **Plasma IL1Beta** | .607^**^ | 0.363 | -0.262 | -0.160 | NC | NC | NC | NC | 0.440 | -0.137 | 1.000 | .744^**^ | -0.295 | -0.087 | -.460^*^ | 0.019 | 0.251 | 0.352 | -.508^*^ | -0.232 | -0.203 |
| **Plasma GDNF** | 0.400 | 0.241 | 0.026 | 0.130 | NC | NC | NC | NC | 0.028 | 0.088 | .744^**^ | 1.000 | -0.328 | -0.231 | -0.114 | -0.125 | 0.099 | 0.083 | -0.319 | -0.234 | -0.252 |
| **Smoking** | -0.103 | 0.047 | -0.137 | -0.195 | -0.395 | -0.250 | -0.212 | -0.232 | 0.065 | -0.371 | -0.295 | -0.328 | 1.000 | -0.054 | -0.197 | -0.031 | -0.233 | -0.016 | 0.103 | -0.012 | 0.098 |
| **Family**  **HX** | 0.029 | 0.307 | 0.060 | 0.091 | -0.212 | -0.347 | -0.077 | -0.145 | -0.154 | 0.115 | -0.087 | -0.231 | -0.054 | 1.000 | -0.034 | 0.159 | -0.043 | -.**365**^*^ | 0.187 | 0.243 | 0.016 |
| **Age** | 0.017 | -0.123 | 0.153 | .**423**^*^ | 0.157 | -0.051 | -0.345 | 0.258 | -0.186 | -0.021 | **-.460^*^** | -0.114 | -0.197 | -0.034 | 1.000 | -0.056 | -0.091 | 0.024 | -0.215 | -.**453**^**^ | -0.226 |
| **Gender** | 0.113 | 0.164 | -.**463**^**^ | -0.037 | 0.292 | 0.399 | 0.107 | .**460**^*^ | 0.115 | -0.346 | 0.019 | -0.125 | -0.031 | 0.159 | -0.056 | 1.000 | .**355**^*^ | -0.175 | -0.010 | -0.104 | 0.206 |
| **PMI** | 0.155 | 0.132 | -0.059 | 0.159 | -0.160 | -0.203 | -0.068 | -0.324 | 0.195 | -0.375 | 0.251 | 0.099 | -0.233 | -0.043 | -0.091 | **.355^*^** | 1.000 | -0.265 | -0.300 | -0.163 | 0.245 |
| **Race** | 0.016 | -0.306 | 0.216 | 0.058 | 0.341 | 0.273 | 0.228 | .**469**^*^ | 0.413 | -0.269 | 0.352 | 0.083 | -0.016 | **-.365^*^** | 0.024 | -0.175 | -0.265 | 1.000 | -0.265 | -0.187 | -0.044 |
| **pH** | -.606^**^ | -0.220 | -0.095 | -0.272 | -.641^**^ | -.**746**^**^ | -0.088 | -0.403 | -.**615**^**^ | **.473^*^** | -.508^*^ | -0.319 | 0.103 | 0.187 | -0.215 | -0.010 | -0.300 | -0.265 | 1.000 | .**751**^**^ | .**376**^*^ |
| **RIN** | -.**439**^**^ | -0.180 | 0.048 | -0.224 | -0.348 | -.**558**^*^ | 0.264 | -.479^*^ | -.**506**^*^ | 0.446 | -0.232 | -0.234 | -0.012 | 0.243 | -.**453^**^** | -0.104 | -0.163 | -0.187 | **.751^**^** | 1.000 | 0.322 |
| **Cause Of Death** | -0.031 | 0.009 | -0.054 | 0.135 | -0.273 | -.**502**^*^ | 0.011 | -0.077 | -0.251 | -0.179 | -0.203 | -0.252 | 0.098 | 0.016 | -0.226 | 0.206 | 0.245 | -0.044 | .**376**^*^ | 0.322 | 1.000 |

**Supplementary Table 3: Levels of interleukins and neurotrophins based on clinical categories in CSF, postmortem-brain and plasma samples.**

|  |  |  | CSF |  |  |  | | | Plasma | |  | |
| --- | --- | --- | --- | --- | --- | --- | --- | --- | --- | --- | --- | --- |
| Group |  | Interleukin/Neurotrophin | | |  | |  | | Interleukin/Neurotrophin | | |  |
|  |  |  | N | Level (pg/ml) | SD(±) | |  | | N | Level (pg/ml) | SD(±) | |
|  | Brain | IL-6 | 10.0 | 67.1 | 67.6 | | | IL-6 | 8.0 | 93.6 | 144.2 | |
|  |  | IL-1β | 10.0 | 220.0 | 122.4 | | | IL-1β | 8.0 | 259.7 | 298.5 | |
| Control |  | BDNF | 10.0 | 231.3 | 94.5 | | | BDNF | 8.0 | 157.1 | 55.5 | |
|  |  | GDNF | 10.0 | 23.8 | 5.5 | | | GDNF | 8.0 | 19.0 | 16.8 | |
|  |  |  |  |  |  | | |  |  |  |  | |
|  | CSF | IL-6 | 10.0 | 480.6 | 458.8 | | | IL-6 | 8.0 | 1663.8 | 1933.1 | |
|  |  | IL-1β | 10.0 | 168.1 | 127.7 | | | IL-1β | 8.0 | 292.8 | 247.7 | |
|  |  | BDNF | 10.0 | 141.8 | 120.9 | | | BDNF | 8.0 | 8525.6 | 2045.6 | |
|  |  | GDNF | 10.0 | 50.5 | 23.0 | | | GDNF | 8.0 | 75.1 | 34.0 | |
|  |  |  |  |  |  | | |  |  |  |  | |
|  | Brain | IL-6 | 6.0 | 84.2 | 118.8 | | | IL-6 | 3.0 | 138.9 | 239.6 | |
| Mood Disorders |  | IL-1β | 6.0 | 204.3 | 153.5 | | | IL-1β | 3.0 | 85.4 | 27.7 | |
|  |  | BDNF | 6.0 | 301.1 | 210.6 | | | BDNF | 3.0 | 169.6 | 162.4 | |
|  |  | GDNF | 6.0 | 39.9 | 12.3 | | | GDNF | 3.0 | 9.2 | 8.3 | |
|  |  |  |  |  |  | | |  |  |  |  | |
|  | CSF | IL-6 | 6.0 | 387.9 | 495.5 | | | IL-6 | 3.0 | 3330.4 | 4770.1 | |
|  |  | IL-1β | 6.0 | 105.8 | 130.8 | | | IL-1β | 3.0 | 217.1 | 159.7 | |
|  |  | BDNF | 6.0 | 82.7 | 69.3 | | | BDNF | 3.0 | 7807.3 | 6437.4 | |
|  |  | GDNF | 6.0 | 34.1 | 11.6 | | | GDNF | 3.0 | 46.2 | 30.8 | |
|  |  |  |  |  |  | | |  |  |  |  | |
|  | Brain | IL-6 | 7.0 | 92.4 | 111.2 | | | IL-6 | 10.0 | 114.0 | 168.8 | |
| Mood Disorder with AUD/SUD |  | IL-1β | 7.0 | 153.8 | 46.8 | | | IL-1β | 10.0 | 197.3 | 174.5 | |
|  |  | BDNF | 7.0 | 278.5 | 194.1 | | | BDNF | 10.0 | 114.9 | 72.5 | |
|  |  | GDNF | 7.0 | 42.7 | 11.2 | | | GDNF | 10.0 | 19.1 | 19.4 | |
|  |  |  |  |  |  | | |  |  |  |  | |
|  | CSF | IL-6 | 7.0 | 887.3 | 1710.5 | | | IL-6 | 10.0 | 1708.6 | 2007.2 | |
|  |  | IL-1β | 7.0 | 109.7 | 75.5 | | | IL-1β | 10.0 | 242.1 | 236.7 | |
|  |  | BDNF | 7.0 | 119.2 | 70.4 | | | BDNF | 10.0 | 8097.1 | 3137.7 | |
|  |  | GDNF | 7.0 | 32.1 | 15.4 | | | GDNF | 10.0 | 37.7 | 27.8 | |
|  |  |  |  |  |  | | |  |  |  |  | |
|  | Brain | IL-6 | 6.0 | 118.8 | 145.4 | | | IL-6 | 7.0 | 72.9 | 98.3 | |
| AUD/SUD |  | IL-1β | 6.0 | 164.8 | 67.4 | | | IL-1β | 7.0 | 121.3 | 83.6 | |
|  |  | BDNF | 6.0 | 250.8 | 101.8 | | | BDNF | 7.0 | 143.8 | 48.3 | |
|  |  | GDNF | 6.0 | 30.2 | 10.6 | | | GDNF | 7.0 | 13.5 | 19.0 | |
|  |  |  |  |  |  | | |  |  |  |  | |
|  | CSF | IL-6 | 6.0 | 968.6 | 1280.5 | | | IL-6 | 7.0 | 3327.3 | 3365.9 | |
|  |  | IL-1β | 6.0 | 104.5 | 155.6 | | | IL-1β | 7.0 | 1116.0 | 2195.0 | |
|  |  | BDNF | 6.0 | 142.0 | 106.0 | | | BDNF | 7.0 | 7144.3 | 3079.3 | |
|  |  | GDNF | 6.0 | 27.5 | 14.5 | | | GDNF | 7.0 | 63.6 | 55.1 | |
