# Supplementary figures and images for: Altered levels of interleukins and neurotrophic growth factors in mood disorders and suicidality: an analysis from periphery to central nervous system
Source: Transl Psychiatry. 2021 Jun 2;11:341. doi: 10.1038/s41398-021-01452-1 (PMC8171230; doi:10.1038/s41398-021-01452-1)

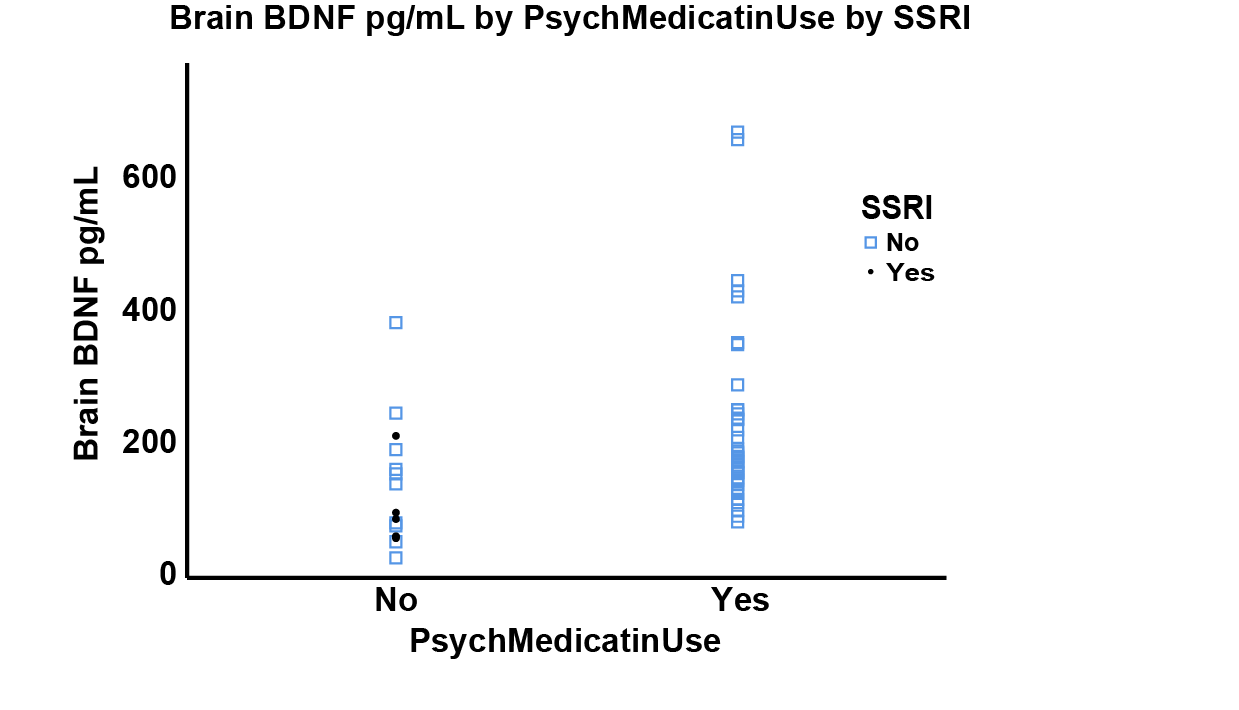

Supplement: Supplementary file 2 — Supplementary Figure-1 [file 41398_2021_1452_MOESM2_ESM.tif]

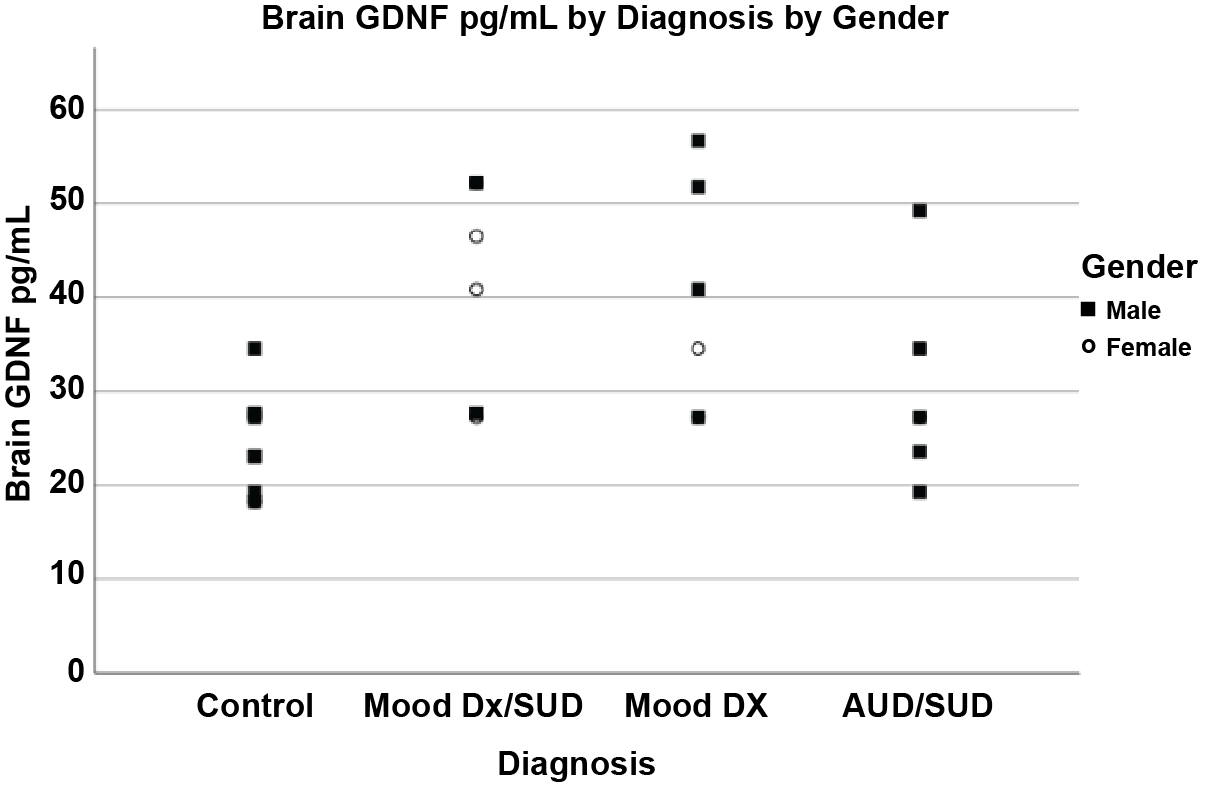

Supplement: Supplementary file 3 — Supplementary Figure-2 [file 41398_2021_1452_MOESM3_ESM.tif]
